# Avian malaria prevalence and mosquito abundance in the Western Cape, South Africa

**DOI:** 10.1186/1475-2875-12-370

**Published:** 2013-10-25

**Authors:** Sharon Okanga, Graeme S Cumming, Phillip AR Hockey

**Affiliations:** 1 Percy FitzPatrick Institute, DST/NRF Centre of Excellence, University of Cape Town, Rondebosch, Cape Town 7701, South Africa

**Keywords:** Avian malaria, Mosquito, Western Cape, South Africa

## Abstract

**Background:**

The close relationship between vector-borne diseases and their environment is well documented, especially for diseases with water-dependent vectors such as avian malaria. Mosquitoes are the primary vectors of avian malaria and also the definitive hosts in the disease life cycle. Factors pertinent to mosquito ecology are likely to be influential to observed infection patterns; such factors include rainfall, season, temperature, and water quality.

**Methods:**

The influence of mosquito abundance and occurrence on the prevalence of *Plasmodium* spp. in the Ploceidae family (weavers) was examined, taking into account factors with an indirect influence upon mosquito ecology. Mosquitoes and weaver blood samples were simultaneously collected in the Western Cape, South Africa over a two-year period, and patterns of vector abundance and infection prevalence were compared. Dissolved oxygen, pH, temperature and salinity measurements were taken at 20 permanent waterbodies. Rainfall during this period was also quantified using remotely sensed data from up to 6 months prior to sampling months.

**Results:**

Sixteen wetlands had weavers infected with avian malaria. More than half of the mosquitoes caught were trapped at one site; when this site was excluded, the number of mosquitoes trapped did not vary significantly between sites. The majority of mosquitoes collected belonged to the predominant vector species group for avian malaria (*Culex culex* species complex). Seasonal variation occurred in infection and mosquito prevalence, water pH and water temperature, with greater variability observed in summer than in winter. There was a significant correlation of infection prevalence with rainfall two months prior to sampling months. Mosquito prevalence patterns across the landscape also showed a close relationship to patterns of rainfall. Contrary to predictions, a pattern of asynchronous co-variation occurred between mosquito prevalence and infection prevalence.

**Conclusion:**

Overall, salinity, rainfall, and mosquito prevalence and season were the most influential vector-related factors on infection prevalence. After comparison with related studies, the tentative conclusion drawn was that patterns of asynchronous variation between malaria prevalence and mosquito abundance were concurrent with those reported in lag response patterns.

## Background

Outbreaks of vector-borne diseases have increasingly been linked to human activities. As people alter landscapes through such activities as forestry, ranching, and agriculture, they may influence disease epidemiology in a variety of ways [1]. In addition to its direct effects on interactions between pathogens and their vectors and hosts, landscape change can alter disease dynamics indirectly via changes in vector ecology. In the case of avian malaria, for example, the influence of host-pathogen relationships on malaria prevalence is fairly well documented [2-4]. However, the impacts of vector ecology and environmental changes on vector ecology are poorly understood. This uncertainty adds a measure of complexity to the prediction of malaria transmission rates.

Mosquitoes are the main vector group for avian malaria [5]. Mosquito abundance is often influenced by environmental factors such as temperature, rainfall, water quality, and habitat [6]. Vector groups for both human malaria (*Anopheles* mosquitoes) and avian malaria (*Culex* mosquitoes, *Culex quinquefasciatus* and *C. univitattus*) demonstrate sensitivity to temperature changes [7]. Further complexity in the epidemiology of avian malaria is introduced by other unknowns, such as the blood meal frequency of infected mosquitoes, transmission rates, and the ratio of vectors to birds in a given habitat [8-10]. Research indicates that anthropogenic activities often alter water quality and availability, and may influence the amount of rainfall that a locality or region receives [11,12]. Such trends will alter the infection patterns of water-borne pathogens and those with water-dependent vectors. The impacts of environmental change on vector-borne diseases may be further enhanced if the vector plays an amplifying role in the pathogen’s life history, as in the case of avian malaria lifecycle, in which sexual reproduction of haemosporidia occurs in the mosquito [5].

Environmental influences on pathogen success have been observed in other host-pathogen systems. For example, the role of water quality is evident in the case of avian influenza prevalence in waterfowl, which co-varies with water salinity [13]. In the lifecycle of *Schistosoma*, snail vectors show faster growth rates when food availability (plant production) is increased in nutrient-enriched waters [14]. Human malaria prevalence is also influenced by water quality, which affects the breeding success of mosquito vectors. Kengluecha *et al. *[15] found that *Anopheles* species abundance fluctuated in accordance with changes in water temperature, pH and dissolved oxygen. The dependence of the mosquito lifecycle on water is strong enough that it can influence patterns of infection at regional extents. For instance, Wood *et al. *[16] showed an apparent pattern of higher infection prevalence in nesting sites of blue tits, *Parus caeruleus,* closer to the River Thames, as a result of increased vector abundance near the water.

The generality of many of these results is unclear. As a test of our emerging understanding of avian malaria ecology, we used a case study in the Western Cape of South Africa to test the following predictions: (1) the infection prevalence of avian malaria would vary with vector abundance; (2) vector abundance and infection prevalence would vary with season and rainfall (with more rainfall encouraging higher prevalence of vectors and avian malaria); and (3) vector type and species would vary with water quality, which would reflect in prevalence patterns.

## Methods

### Sampling sites

Research was conducted after approval from the Science Faculty Animal Ethics Committee, University of Cape Town and carried out in strict accordance with the recommendations given by the committee. Research did not involve the sampling of endangered or protected species. Access to field sites was granted by private landowners in the Western Cape and the City of Cape Town. Research permits granting access to protected areas were issued by SANParks (South African National Parks Board) and by Cape Nature (the Western Cape Nature Conservation Board).

Sampling was conducted at 20 perennial wetlands of 1 – 10 hectares in size, in the Western Cape Province of South Africa [17]. Wetlands were chosen as study sites, as they are resource-rich and act as key habitats for a large variety of birds [18]. All sites were located between altitudes of 0 – 300 m above sea level. In the Western Cape, summer occurs in the months of January to March, and winter in the months of July to September. Unlike other parts of South Africa, the Cape region experiences winter rainfall. The Cape is currently devoid of human malaria, but has a history of avian malaria infection [19-21]. Sites were visited once per year for two years (between 2010 and 2011). Samples were collected during each visit; visits were timed to ensure that each site was visited once during summer and once during winter, with samples collected during each visit.

### Sampling of birds

Birds from the Ploceidae family (bishops, weaver birds, and allies - hereafter referred to as 'weavers’) were the target group and were trapped using mist nets. Although weavers can be highly mobile, the species trapped were mostly residential [22]. In keeping with their social nature, weavers tend to move *en masse* and live in nests built close together, and situated over water surfaces [22]. It is, therefore, quite likely that infected birds caught at a particular site were infected at the same sampling site.

Birds were sampled by pricking the brachial vein and collecting blood into a capillary tube [23], which was then preserved in vials containing lysis buffer. The vial was sealed and the sample sent for molecular processing. All birds were ringed (to identify potential recaptures) and released after sampling.

### Molecular analysis

Blood samples were analysed using PCR, as detailed by Cumming *et al. *[24]. MEGA 5.0 (Molecular Evolutionary Genetics Analysis [25]) was used to conduct genetic analyses, to choose the most appropriate model of evolution, and to construct maximum likelihood (ML) phylogenetic trees. In addition, a Bayesian analysis was run in MR Bayes for 1 million generations with a burn-in of 250 000 generations, sampling one in every 100 trees [26,27]. The general time reversible model with a discrete gamma distribution (GTR + G; G = 0.20) received the best Bayesian Information Criterion score and was applied to the ML Bayesian phylogenetic analysis. Node support was evaluated using 1,000 bootstrap replicates, with bootstrap values greater than 50% used for the final tree [28]. Sample sequences emerging in the same branches as GenBank sequences were assumed to be the same species and in this study are collectively referred to as a 'clade’. Seven individual *Plasmodium* spp. lineages were isolated altogether, with clades numbered from I-VII. Two dual infection types were also isolated and were represented with the numbers of each clade causing infection.

### Mosquito sampling

Mosquitoes were trapped using two CDC miniature light traps (model 512, John W. Hock company). The traps were placed at opposite ends of the wetland, or at least 200 m from each other, to avoid overlap in the areas being sampled. Traps were suspended approximately 1.5 metres above ground, from a tree or bush and placed at a distance not exceeding 20 m from the water’s edge. Traps were operated using a 6 volt lead-acid motor cycle battery, which would run a trap for two nights when fully charged. Traps were placed out from dusk until dawn in 12 hour trapping cycles, after which they were retrieved. Trapping sessions consisted of one to two nights per site, depending on the numbers of mosquitoes trapped and the success of the trap location. Mosquitoes in the trap were individually removed and placed into a 10 ml vial containing absolute ethanol.

In the laboratory, vials were decanted into a petri dish with filter paper. Mosquitoes were separated from the other insects caught in the trap, and left to dry on a separate piece of dry filter paper. Dried mosquito specimens were individually examined and identified using a Nikon SMZ-10 stereomicroscope. Identification of mosquito specimens was facilitated by the use of a handbook detailing local South African species and their distributions [29]. This handbook is specific only to the identification of female mosquitoes in the Culicinae and Toxorhynchitinae families. *Anopheles* mosquitoes and male culicine specimens were sent for identification to the VCRU (Vector Control Reference Unit) at the NCID (National Institute for Communicable Diseases, Johannesburg, South Africa), together with specimens that could not be identified using the handbook. Mosquito species were noted as potential vectors in accordance with Russell and Mohan [30], Njabo *et al. *[31] and Ventim *et al. *[32].

### Water quality

Water quality was measured at each wetland site using an HI 9828 Hanna handheld water meter (HANNA instruments). Water samples were taken from three stations situated at opposing points around the wetland and sampled consistently at the same points during all sessions. Measurements recorded were dissolved oxygen, temperature, pH and salinity. Measurements were made every two metres into the water body, starting at 1 m from the shoreline up to a distance of 20 m, or as far into the waterbody as depth would allow. Sampling was conducted once during summer and once during winter for each site.

### Rainfall

Rainfall data were obtained from the FEWS (Famine Early Warning Systems) net portal [33]. The site provides remote spatial data for various regions worldwide, including estimates of daily rainfall (RFE). RFE is calculated using a rainfall estimation algorithm that incorporates cloud top temperature and rainfall data from various stations acquired at six-hour intervals at a resolution of 0.25 degrees [34]. RFE data were downloaded from the southern Africa region files available on the site.

The daily rainfall data were summed to give a monthly rainfall estimate (MRFE), as well as seasonal and annual rainfall estimates for each site. MRFEs, together with the number of days of rainfall per month, were calculated for up to six months prior to the sampling month (i.e. over the period of July 2009 to September 2011). This was done in accordance with the results of Mbogo *et al.*[35], who showed that vegetation, mosquito abundance and infection prevalence display a lag response to rainfall patterns, and that rainfall (and days of rainfall) from preceding months can potentially influence disease infection prevalence.

### Statistical analysis

All statistical analysis was conducted in R (2011_12_22) [36]. Samples were ordered by season collected (winter or summer). Readings from all sampling stations were collated to generate a mean value for each parameter measured per site for each season, and all parameters were used in analysis.

All variables were tested for normality using the Shapiro-Wilkes test. Only salinity was non-normal. To reduce heteroscedasticity in models [37], salinity data were log-transformed using a Box-Cox transformation [38], dividing by the mean and adding a constant as per the following equation:

(1) $$\text{salinity}\prime =\log\left(\text{salinity}/\text{mean}\left(\text{salinity}\right)+k\right)$$

where k is a constant > 0.

Because water quality tends to display spatial heterogeneity in accordance with varying altitude and geology in a landscape [39], the longitudinal and latitudinal values of each wetland site were also included as explanatory parameters in multiple regression analysis.

Some mosquito specimens were unidentifiable due to immaturity and key body parts missing, and were not included in analysis. Mosquito data were quantified in two ways: mosquito prevalence and vector abundance. Mosquito prevalence was calculated as the sum of mosquitoes caught per site divided by total number of mosquitoes caught overall. For sites where trapping was conducted over two nights, the mean number of mosquitoes caught was calculated to provide comparability with sites where mosquitoes were trapped over one night.

As data for vector species for avian malaria are still quite sparse for the Western Cape region, there remains the possibility that additional undocumented species act as vectors. We simply used the abundance of known vectors to gauge vector presence. Vector abundance was thus the number of known vectors caught per site.

A disproportionate number of mosquitoes were caught at one wetland site. Because trapped mosquito samples displayed a non-normal distribution, the inter-quartile range of the dataset was employed as an outlier identification method applicable to non-normally distributed data [40], to identify outliers as per the following:

(2) $$\text{Outlier}>1.5\left(Q_{3}-Q_{1}\right)$$

And extreme outliers as:

(3) $$\text{Extreme}\;\text{Outliers}>\left(Q_{3}-Q_{1}\right)$$

Where Q_1_ = 1^st^ quartile; Q_3_ = 3^rd^ quartile.

The number of mosquitoes caught at this wetland site qualified as an extreme outlier in the dataset. Consequently, mosquito abundance data were analysed with and without the inclusion of this site in the data, to compensate for potential distortion in regression analyses.

Simple and multivariate regression was used to explore the relationships between infection prevalence and mosquito/vector prevalence, water quality and vector prevalence, and water quality and infection prevalence. Pearson’s product–moment correlations (*r*^*2*^) were run between infection prevalence and water quality elements, infection prevalence and mosquito/vector abundance; and mosquito/vector abundance and water quality. Significant variation within and between water quality parameters, seasonal infection prevalence, and the number of mosquitoes caught was tested using Chi-squared test, Welch’s two tailed *t*-test and ANOVA.

Multiple regression analyses were run using general linear models (GLMs) fitted with Poisson (canonical) links corresponding to the nature of the prevalence distribution. Model selection was conducted using Akaike’s Information Criterion value (AIC) as the indicator of the best-fit model. Model selection was conducted using values from Akaike’s information criterion (AIC) as an indicator of the best-fit model, with predictor variables retained or removed depending upon their effect upon model AIC value.

## Results

### Overall results

From 20 sites, 150 (27%) weavers out of 580 sampled birds were infected with *Plasmodium* spp.; only 4 sites had no infected birds. 524 mosquitoes were caught from 16 sites, with 345 (66%) caught at Strandfontein (STR). Mosquitoes trapped did not vary significantly between sites, after analysis including data from STR (*F*_1, 24_ = 0.07) and excluding STR (*F*_1, 24_ = 0.27).

### Seasonal variation in infection prevalence and lineage prevalence

**Figure 1:**
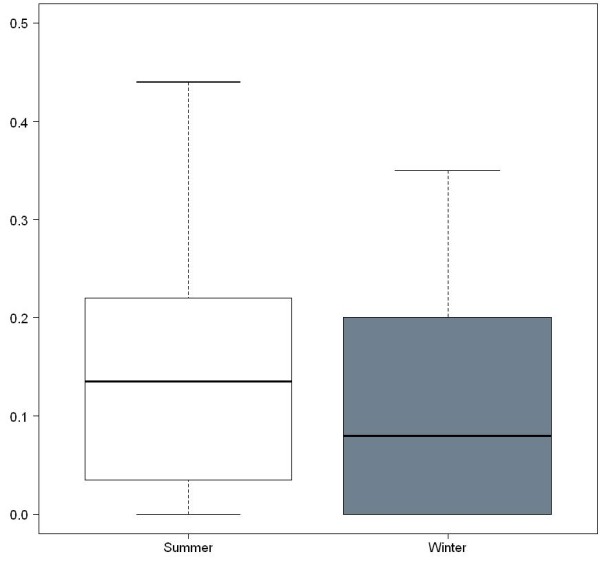
**Seasonal prevalence of *Plasmodium *** **infections amongst weaver birds.**

**Figure 2:**
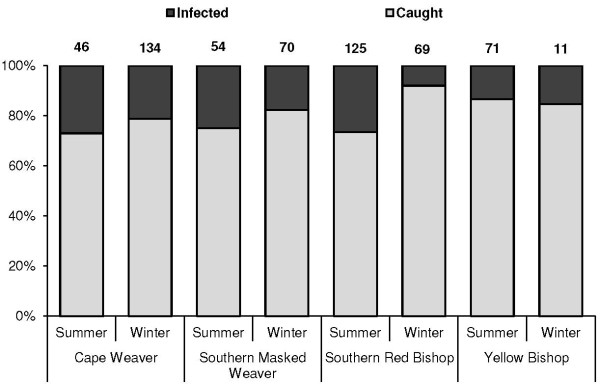
**Seasonal infection prevalence of infected weavers.** The number of birds caught per species is indicated above each column (n = 580).

Out of the total sampled population, ninety-one weavers (16%) were infected during summer sampling months and 59 weavers (10%) were infected during winter sampling. Overall infection prevalence displayed significant seasonal variation (Figure 1; *x*^2^ = 180; d. f. = 81; *p* < 0.001). Southern Red Bishops were the most abundant weaver species caught and also were the most heavily infected weaver species, with 26% of caught birds infected (Figure 2). Southern Red Bishops were also the only species to display significant variation in infection prevalence between seasons (*x*^*2*^ = 21.86; *p* < 0.001), with most birds infected during the summer.

### Variation in lineage prevalence

**Figure 3:**
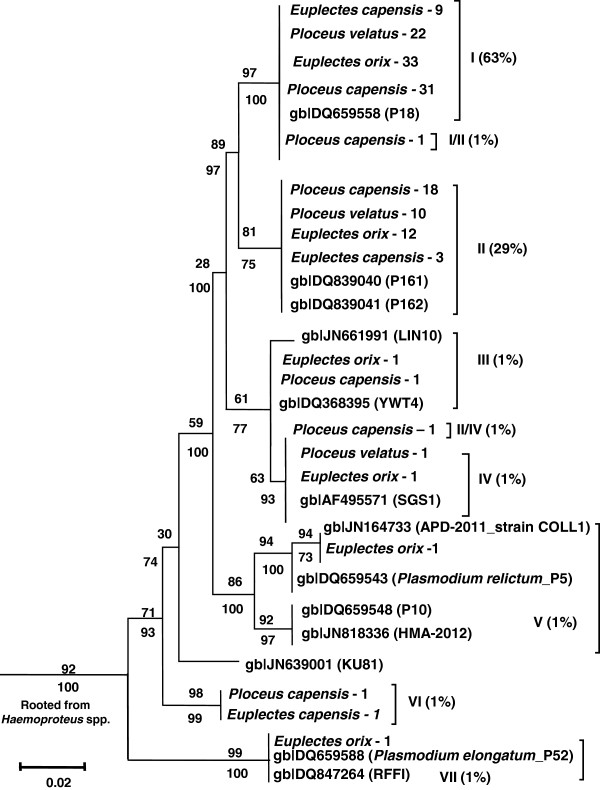
**Plasmodium phylogenetic tree with bootstrap values (> 50%) displayed.** Nodal support values from maximum likelihood analysis are displayed above branches and those from Bayesian analysis are below. Letters identify the clade (n = 9) with clade infection prevalence in brackets (n = 150). Weaver species infected are *Ploceus capensis* (Cape Weaver), *Ploceus velatus* (Southern Masked Weaver), *Euplectes orix* (Southern Red Bishop) and *Euplectes capensis* (Yellow Bishop). Matching or closely related lineages from Genbank are also displayed in the tree.

**Figure 4:**
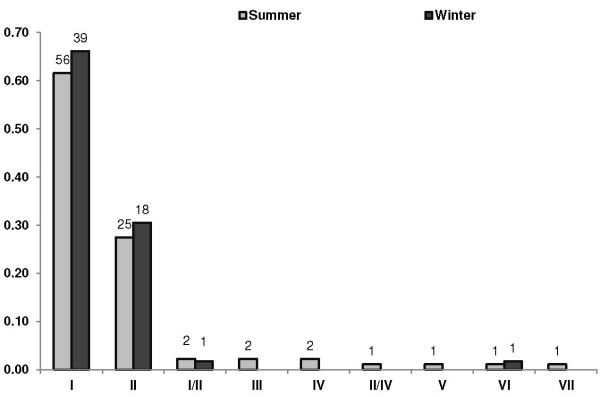
**Seasonal infection prevalence of *Plasmodium *lineages.** The number of birds infected per lineage is indicated above each column (n = 150).

Clade I was the predominant lineage occurring throughout both seasons. It infected more birds than any other lineage, and also occurred in one of two dual infections observed (Figure 3). Two birds also displayed dual infections. *Plasmodium* lineages mainly occurred during the summer, with only four lineages also occurring during winter (Figure 4). However, no significant difference was found in prevalence for lineages occurring both in summer and winter (*t* = 0.46; d. f. = 14.48; *p* = 0.65).

### Variation in mosquito and vector abundance

Fifteen species from five genera were identified from 153 specimens (40% of the total catch), as listed in Table 1. Five of these species, from the *Culex* genus and *Culiseta* genera, are known vectors of avian malaria.

**Table 1:** Mosquito species caught across sites (n = 524)

**Mosquito species**	**Sites present**	**Summer**	**Winter**	**Total catch**
***Culex Culex***				
*Culex culex pipiens* complex^$^*	9	44	2	46
*C. c. theileri* *	9	20	5	25
*C. c. univitattus* *	6	14	6	20
*C. c. decens*	4	6	0	6
*C. c. chorleyi*	2	3	0	3
*C. c. andersoni bwana*	1	1	0	1
*C. c. simpsoni*	1	1	0	1
**Total**		**92**	**13**	**105 (67%)**
***Culex culiciomyia***				
*C. culiciomyia cinerellus*	1	1	0	1
**Total**		**1**	**0**	**1 (0.6%)**
***Culex eumelanomyia***				
*C. eumelanomyia inconspicuosus*	4	6	1	7
*C. e. simpliforceps*	6	16	1	17
**Total**		**22**	**2**	**24 (15%)**
***Culiseta***				
*Culiseta allotheobaldia longeareolata* *	8	8	2	10
**Total**		**8**	**2**	**10 (6%)**
***Anopheles***				
*Anopheles coustani*	1	11	0	11
*A. squamous*	1	1	1	2
**Total**		**12**	**1**	**13 (8%)**
***Toxorhyncites***				
*Toxorhyncites toxorhyncites brevipalpis*	2	2	0	2
**Total**		**2**	**0**	**2 (1%)**
***Aedes***				
*Aedes aedimorphus albocephalus*	1	1	0	1
**Total**		**1**	**0**	**1 (0.6%)**
**Unknown (unidentifiable/missing parts/immature)**				
		**352**	**16**	**368**
**Grand Total**		**490 (94%)**	**34 (6%)**	**524**

Names in bold indicate the mosquito genera identified. Generic prevalence is indicated in brackets; known vectors are denoted by *. ^$^The *Culex culex pipiens* complex includes *Culex c. pipiens* and *C. quinquefasciatus* which are often indistinguishable from each other.

**Figure 5:**
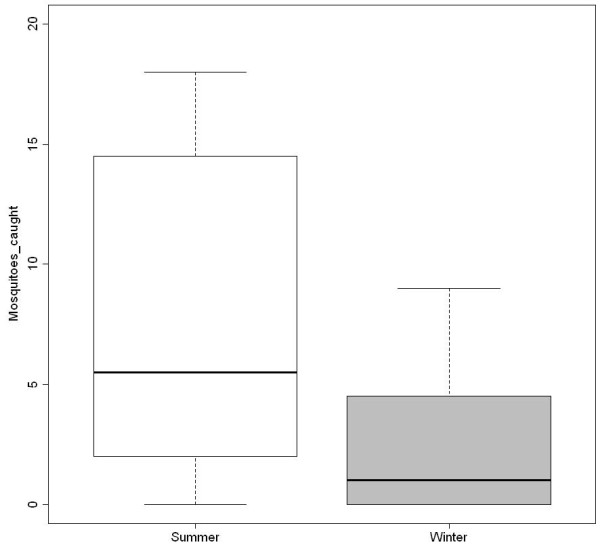
**Seasonal variation in the mean number of mosquitoes caught per site (n = 524; *p*** **= 0.04).**

Mosquito abundance varied significantly between seasons (Figure 5): 490 individuals (between 1–260 specimens per site) were caught in the summer from 14 sites, whereas only 34 (1 – 8 specimens per site) were caught in winter from eight sites (*t* = 2.13_1, 38_; *p* = 0.04). Overall, *Plasmodium* infection per site showed little correlation with vector abundance (*r*^*2*^ = - 0.29; *p* = 0.21). Mosquito seasonal prevalence and *Plasmodium* infection were not significantly correlated (*r*^*2*^ = - 0.28; *p* = 0.08), although this association strengthened notably after the exclusion of STR mosquitoes (*r*^*2*^ = - 0.33; *p* = 0.04).

### Water quality, infection and vector prevalence

Seasonal variation also occurred in water quality elements measured (Table 2). Temperature varied clearly from summer to winter (*t* = 11.98_1, 33.74_; *p* < 0.001), with higher temperatures occurring in summer. There was significant variation in pH values (*t* = 2.09_1, 36.56_; *p* = 0.04), with increasing alkalinity observed during winter.

**Table 2:** Seasonal variation in sampled water quality variables

	**Dissolved oxygen**	**Temperature**	**pH**	**Salinity**
Summer range	2.97 – 11.54	17.29 – 33.22	5.40 - 9.31	0.00 – 6.52
Summer mean	7.02	**27.28**	**7.94**	0.77
Winter range	2.07 – 14.02	10.72 – 19.20	6.85 – 10.11	0.00 – 2.18
Winter mean	7.76	**14.80**	**8.43**	0.63

Numbers in bold indicate significant seasonal variation, which occurred with temperature (*p* < 0.001) and pH (*p* = 0.04).

Only salinity was correlated with infection prevalence (*r* = 0.39; d. f. = 38; *p* < 0.03), with *Plasmodium* prevalence increasing with rising salinity values (see Table 3). There was also significant correlation between vector abundance and temperature (*p* = 0.02); and between mosquito prevalence and dissolved oxygen (*p* = 0.05). Dissolved oxygen and pH were positively correlated with each other (*r*^*2*^ = 0.52; *p* = 0.02), as were salinity and latitude (*r*^*2*^ = 0.39; *p* < 0.01).

**Table 3:** **Values for Pearson’s coefficient (*r***^***2***^) describing co-variation between water quality elements,*Plasmodium*and vector abundance (*p **≤ 0.05)**

	**Temp**	**pH**	**DO**	**Salinity**	**Latitude**	**Longitude**
***Plasmodium *** **prevalence**						
	- 0.21	0.10	0.18	0.39*	0.33*	- 0.13
**Mosquitoes**						
Mosquito prevalence/WSTR	0.09/- 0.12	0.008/0.28	0.10/0.44*	- 0.33/- 0.33	- 0.16/- 0.23	- 0.13/0.27
Vector abundance	0.37*	- 0.11	0.003	- 0.16	- 0.17	- 0.05

WSTR represents mosquito prevalence excluding Strandfontein. Temp = Temperature; DO = Dissolved oxygen. Numbers in bold indicate significant correlation.

### Variation in infection prevalence with rainfall and location

**Figure 6:**
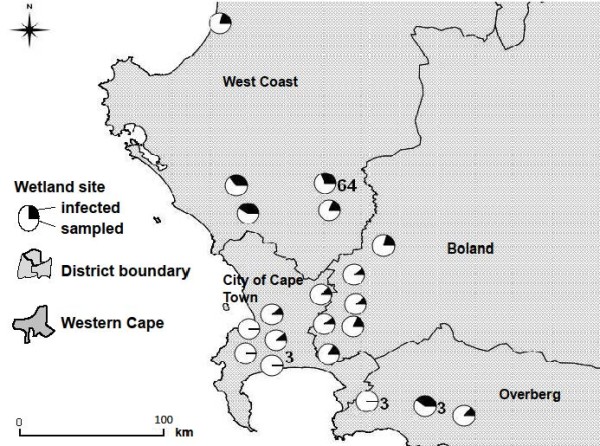
**Map showing infection prevalence by site (overall site prevalence = 0.27; n = 20).** Numeric labels indicate sites with the smallest and largest sample sizes. There was a significant pattern of variation in *Plasmodium* prevalence with location (*p* < 0.001; see Table 2), with most heavily infected sites situated in the West Coast District.

*Plasmodium* prevalence varied significantly across districts (see Figure 6; *F*_3, 15_ = 8.21; *p* = 0.002). Sites in the west coast district displayed infection prevalences of 23 – 70% (mean 44%; s. d. = 9.56). The west coast district was the driest region from which birds were sampled, with sites in that region receiving between 200–400 mm rainfall annually. Sites in the wetter districts of the City of Cape Town and Boland (which received between 600–800 mm rainfall) had lower mean infection prevalences of 7% and 12% respectively. *Plasmodium* prevalence was also correlated with latitude (*p* = 0.04; see Table 3).

**Figure 7:**
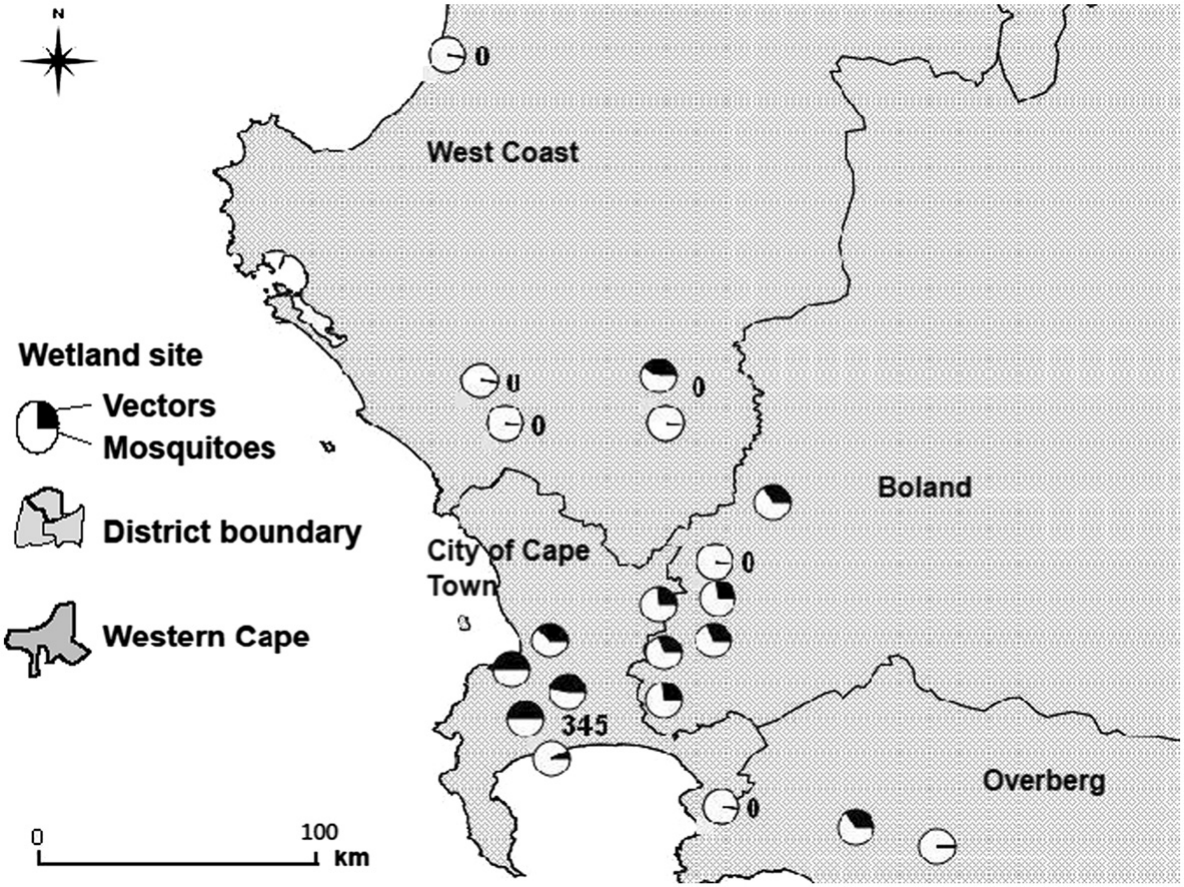
**Map displaying prevalence of mosquitoes and potential vectors caught per site (total catch = 516; n = in 20 sites).** Numeric labels indicate sites with the smallest and largest sample sizes; notably, no mosquitoes were caught at 6 sites. The most mosquitoes were trapped in the Boland and Cape metropolitan districts.

With the exception of Strandfontein (which falls within the City of Cape Town), the largest number of mosquitoes were caught in and around the Boland District, whereas the lowest number were caught in the West Coast District (Figure 7). This variation in mosquitoes caught across districts was notable, although not significant (*F*_3, 15_ = 3.03; *p* = 0.06).

**Figure 8:**
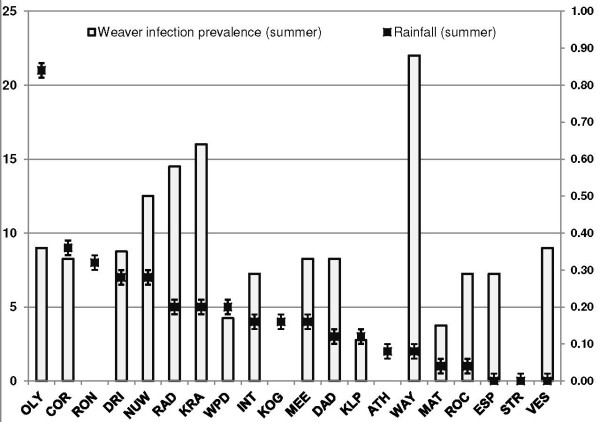
Rainfall (mm, left axis) and site prevalence (right axis) in summer sampling months (standard deviation bars = +/- 0.49).

**Figure 9:**
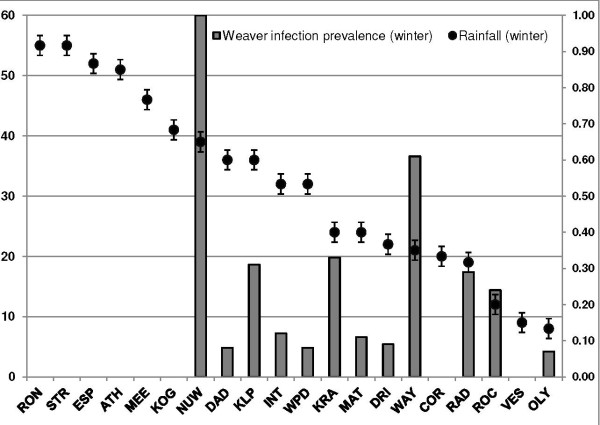
**Rainfall (mm, left axis) and site prevalence (right axis) in winter sampling months (standard deviation = +/- 1.65).** The pattern of higher infection prevalence with less rainfall is more apparent during winter.

Variation in infection prevalence with rainfall followed a seasonal pattern, with wetter sites generally displaying lower infection prevalences (Figures 8 and 9). Infection prevalence showed significant correlation with rainfall (*r*^*2*^ = - 0.43; *p* = 0.05) and days of rainfall (*r*^*2*^ = - 0.49; *p* = 0.02) two months prior to sampling across seasons. Seasonally, infection prevalence was negatively related to rainfall during winter sampling months (*r*^*2*^ = - 0.43; *p* = 0.05) and days of rainfall 2 months prior (*r*^*2*^ = - 0.51; *p* = 0.02).

### Multiple regression analysis

*Plasmodium* prevalence was best described by seasonal factors (with winter as the significant season); mosquito prevalence; salinity; and rainfall at a lag of 4 months (Table 4). Rainfall during the sampling month was also a relevant factor, although not as influential. Significant reductions in the goodness of fit of the best model occurred when any explanatory variables were omitted. Season caused the greatest reduction when omitted (likelihood ratio = 17.47; *p* < 0.001) and rainfall (at four months) the least reduction (likelihood ratio = 4.16; *p* = 0.04).

**Table 4:** **Models best describing** ***Plasmodium*** **prevalence (n = 20)**

**Factor**	**Coefficient estimate (± S.E.)**	***P***	**d.f.**	**Residual deviance**	**AIC**	**ΔAIC**
	*Plasmodium* (best-fit model)					
**Intercept**	- 0.86 ± 0.17	<0.001	34	69.37	179.54	**-**
**Winter**	- 0.78 ± 0.18	<0.001				
**Salinity**	0.20 ± 0.05	<0.001				
**Mosquito prevalence**	- 5.85 ± 2.00	<0.005				
**Rainfall (at 4 months)**	- 0.01 ± 0.008	<0.05				
	*Plasmodium* model 2					
**Intercept**	- 1.13 ± 0.11	<0.001	36	75.53	181.70	2.16
**Winter**	- 0.58 ± 0.16	<0.001				
**Salinity**	0.25 ± 0.05	<0.001				
**Mosquito prevalence**	- 6.80 ± 1.99	<0.001				
	*Plasmodium* model 3					
**Intercept**	- 1.10 ± 0.11	<0.001	35	72.80	182.97	3.43
**Winter**	- 0.41 ± 0.25	<0.001				
**Salinity**	0.25 ± 0.05	<0.001				
**Mosquito prevalence**	- 6.55 ± 2.01	<0.001				
**Rainfall (sampling month)**	- 0.007 ± 0.009	0.40				
	*Plasmodium* model 4					
**Intercept**	- 1.01 + 0.16	<0.001	36	74.17	184.89	5.35
**Salinity**	0.22 + 0.06	<0.001				
**Mosquito prevalence**	- 5.46 + 1.97	0.006				
**Rainfall (sampling month)**	- 0.02 + 0.01	<0.005				
**Rainfall (at 4 months)**	- 0.01 + 0.01	0.32				
	*Plasmodium* model 5					
**Intercept**	- 8.50 ± 0.17	<0.001	36	80.72	188.90	9.36
**Winter**	- 0.86 ± 0.18	<0.001				
**Salinity**	0.21 ± 0.06	<0.001				
**Rainfall (at 4 months)**	- 0.02 ± 0.01	<0.005				

Models are ranked using ΔAIC and range between ΔAIC ≤ 10.

## Discussion

*Plasmodium* infections were widespread across sampling sites, with only four sites having no infected birds. The majority of mosquitoes (66%) were caught at one wetland, Strandfontein (STR). Strandfontein was amongst the sites receiving the highest annual rainfall (503 mm), which was likely a primary contributing factor to the high mosquito catch. Strandfontein is also a sewage treatment plant, and it is possible that the high nutrient content of the water enhanced mosquito breeding success at this site [41,42]. However, because no nutrient data were collected in this study, this suggestion remains open.

Temperature and pH displayed the most marked variations between seasons (Table 2), which is consistent with previous findings [43,44]. Temperature varied with vector abundance, which was also consistent with previous findings on associations between water quality and malaria vectors [10,15]. These elements did not display any apparent influence on either mosquito or *Plasmodium* prevalence (see Figures 1 and 5). Otherwise prevalence patterns were synchronized across seasons, with higher prevalences seen in summer than in winter (Figures 2, 4 and 5). Seasonal factors tend to have contrasting effects upon different vector species; seasonal variation in the prevalence of vector-borne diseases is well documented [45], and our observed patterns are in accordance with findings from other studies of avian malaria [46,47], as well as other vector-borne diseases [48].

Sites with the heaviest infection prevalence were also the sites with the lowest mosquito catch. Three (out of five) sites with heavy infections were situated in the west coast district of the Western Cape; no mosquitoes were caught at these sites, despite an equal sampling effort across all sites (Figures 6 and 7). Because mosquitoes are dependent upon water for breeding it is expected that they would be more abundant in wetter areas. Mosquito catch size across sites did indeed vary with rainfall patterns (Figure 7), which is supportive and consistent with previous findings [49,50]. The West Coast District is much drier than other sampled districts in the province [51], receiving ≤ 200 mm of rainfall annually compared to sites within the Cape Town metropole (≥ 700 mm), and sites in the Boland and Overberg Districts (400 – 600 mm). The western coast of the Western Cape is one of the driest regions in South Africa, with climate modelling indicating that it will become still drier over the coming years [52]. Observed seasonal patterns of infection and mosquito abundance are in contrast to patterns observed elsewhere in the country [53], most likely because the Western Cape is a winter-rainfall region, unlike the rest of South Africa. Rainfall had both a direct and indirect impact upon infection prevalence (Figures 8 and 9), and also featured in models explaining variation in prevalence (Table 4).

Seasonal abundance was the only apparent pattern of analogous co-variation between mosquito and infection prevalence. Otherwise, the notable outcome was the contrasting relationship between mosquito and infection prevalence. The two factors displayed a negative correlation with each other, although the expectation was that they would co-vary positively. The negative co-variance of mosquito prevalence and infection prevalence is apparently concurrent with observations from previous malaria studies in Africa, which report lag responses in mosquito abundance and infection prevalence, both with each other and with rainfall [9,35]. Similar studies in Africa also found that at several sampling sites with high infection prevalences (of *Plasmodium falciparum*), few to no mosquitoes were caught [35,54]. Additionally, the entomologic inoculation rate was low in comparison to infection prevalence, and varied widely in small geographic regions of sampling.

Taken in context, the gathered data thus suggest that malarial infection prevalence is not dependent upon current mosquito abundance - leading to three parsimonious conclusions. The first is that the patterns of prevalence observed here are indicative of malarial and mosquito prevalence exhibiting a lag response throughout the year in response to the same driving factors. This potentially explains the contrasting relationship between the two prevalences, and the observed correlations with rainfall in the months prior to sampling. The second conclusion is that a low number of mosquitoes are still capable of infecting a wide range of hosts. The third conclusion is that birds were not infected at the wetlands at which they were caught. As the majority of sampled birds were territorial wetland passerines, and predominantly residential [22], it was assumed that these birds were infected on site. However, there remains the possibility that species sampled were more mobile than current knowledge suggests – potentially leading to a scenario where birds were caught at one wetland but actually infected at another location. This possibility is further complicated by the fact that wild passerines can carry chronic lifelong infections of avian malaria, which can have various effects (depending on the host species) on host mobility and reproductive success [55,56]. For example in this study, a significant variation in species infection prevalence with season occurred only in Southern Red Bishops, but not with other sampled weaver species (Figure 2). Incorporating movement data (of sampled bird species) into future analyses would serve to confirm the significance of host movement in observed avian malarial infections. Also of interest was that many wetland sites with a low mosquito catch had exposed banks with little to no vegetation within the set trapping areas. Water with high detrital loads (from erosion or a similar event) is not conducive to mosquito breeding, and these wetlands may have acted as a deterrent to mosquito breeding [57]. Leisnham *et al. *[57] similarly noted that the level and type of vegetation cover mattered in mosquito breeding success. Hence mosquito prevalence here was potentially further affected by variation in vegetation cover and type, or changing landscape features. A final factor for consideration is that several mosquito species caught in this study may have been vectors for avian malaria, but are not yet known as vectors; leading to a potential underestimation of vector abundance. The fairly recent reports of new vector species in Africa [31] highlight how limited vector knowledge is pertaining to avian malaria. Consequently, our conclusions are restricted by the lack of current knowledge about known vectors for avian malaria, particularly in sub-saharan Africa.

Salinity was the only element of water quality that exhibited a direct correlation with *Plasmodium* infection prevalence (Table 3). Infection also varied with latitude, whereas salinity and latitude were significantly and positively correlated (*r*^*2*^ = 0.39; *p* = 0.01). The trend in infection prevalence was apparent across sampling regions (Figure 6), suggesting that location is an influential factor in both wetland salinity and infection prevalence. The link between salinity and *Plasmodium* may be an effect of *Plasmodium* prevalence being higher in the summer, when waterbodies tend to be more saline as a result of increased evaporation [58,59]. It was also expected that salinity would increase with proximity to the sea – instead the opposite trend was observed. The locational trend in salinity variance implies there is an additional underlying factor influencing water quality, such as the geology of the landscape within which the wetland occurs. For instance, several sites in the West Coast District occurred in areas typified by geology such as shales and batholiths, which occur around Malmesbury and Darling respectively [60]. The chemistry of these volcanic rocks [60,61], combined with the land use of the surrounding areas [17], may subsequently influence water quality.

*Plasmodium* prevalence was best described by a model incorporating locational and seasonal factors together with salinity, rainfall and mosquito prevalence (Table 4). With the exception of mosquito prevalence, these factors were all influential to infection prevalence. Mosquito prevalence exerted an indirect influence in the *Plasmodium* model, further suggesting that the relationship between infected birds and mosquito prevalence is non-linear, and supporting the argument that mosquito and infection prevalences vary temporally in a lag pattern with each other.

## Conclusions

The prevailing outcome of this analysis was that avian malaria prevalence and mosquito abundance did not display analogous co-variation. Instead, they varied in a contrasting fashion and were indirectly linked by season and rainfall. The presence of infected birds at a site typically indicates the presence of a vector species; however, at many infected sites (particularly in the West Coast district of the Western Cape) no mosquitoes were caught. The same result has been reported in previous malarial studies [35,54], which also demonstrated temporal lag responses between mosquito prevalence, infection prevalence and rainfall. The best-supported explanation for observed prevalence patterns is a probable lag response between vector prevalence and rainfall. Another trend was that local environmental variation played a prominent role in avian malarial infection prevalence in the Western Cape. In general, however, the findings presented here support the argument that avian malaria prevalence patterns vary with both host and vector-related factors.

## Competing interests

The authors declare that they have no competing interests.

## Authors’ contributions

SO contributed to the design of the study; conducted fieldwork, mosquito identification and data analysis; and wrote the first draft of the manuscript. GC contributed to the design of the study, data analysis, and editing. PH contributed to the design of the study and editing. PH passed away before the manuscript was finalized. All authors have read and approved the final manuscript. 
